# Clustered Distributed Data Storage Repairing Multiple Failures

**DOI:** 10.3390/e27030313

**Published:** 2025-03-17

**Authors:** Shiqiu Liu, Fangwei Ye, Qihui Wu

**Affiliations:** 1 Pengcheng Laboratory, Shenzhen 518066, China; 2 College of Computer Science and Technology, Nanjing University of Aeronautics and Astronautics, Nanjing 211106, China; 3 Key Laboratory of Dynamic Cognitive System of Electromagnetic Spectrum Space, Ministry of Industry and Information Technology, Nanjing University of Aeronautics and Astronautics, Nanjing 211106, China; wuqihui2014@sina.com

**Keywords:** clusteredstorage system, regenerating code, minimum storage, minimum bandwidth

## Abstract

A clustered distributed storage system (DSS), also called a rack-aware storage system, is a distributed storage system in which the nodes are grouped into several clusters. The communication between two clusters may be restricted by their connectivity; that is to say, the communication cost between nodes differs depending on their location. As such, when repairing a failed node, downloading data from nodes that are in the same cluster is much cheaper and more efficient than downloading data from nodes in another cluster. In this article, we consider a scenario in which the failed nodes only download data from nodes in the same cluster, which is an extreme and important case that leverages the fact that the intra-cluster bandwidth is much cheaper than the cross-cluster repair bandwidth. Also, we study the problem of repairing multiple failures in this article, which allows for collaboration within the same cluster, i.e., failed nodes in the same cluster can exchange data with each other. We derive the trade-off between the storage and repair bandwidth for the clustered DSSs and provide explicit code constructions achieving two extreme points in the trade-off, namely the minimum storage clustered collaborative repair (MSCCR) point and the minimum bandwidth clustered collaborative repair (MBCCR) point, respectively.

## 1. Introduction

Cloud distributed storage systems (DSSs), built on a huge number of storage nodes, have been applied widely (e.g., Google File System [1], Facebook [2], and Total Recall [3]). In a DSS, failures occur frequently, degrading the system’s reliability. To ensure the reliability of DSSs, erasure coding has been widely used to improve their tolerance of failures.

A class of erasure codes, namely *regenerating codes*, for cloud distributed storage systems was proposed by Dimakis et al. [4], aiming to reduce the repair bandwidth (or repair traffic) during node failures. More specifically, suppose that a data file is encoded and stored on *n* nodes, each of which stores α symbols. A data collector can retrieve the data file by connecting to any *k* nodes, which is called the *(n,k) recovery* property. When a node failure occurs, the system will trigger a repair process to recover the data on the failed node by downloading data from some other nodes. The total amount of data downloaded from these other nodes is called the repair bandwidth, which consists of γ symbols. The fundamental trade-off between the amount of data stored on each node α and the repair bandwidth γ was studied in [4]. There are two extreme points on the trade-off curve, namely the minimum storage repair (MSR) point and the minimum bandwidth repair (MBR) point, which minimize the data stored on each node and the repair bandwidth, respectively.

The pioneering work [4] proposed studying a single failure in a cloud distributed storage system. Later, *collaborative regenerating codes* (CRCs) were proposed [5,6,7] for studying cases when multiple failures happen and are repaired simultaneously in a DSS, which has been shown to improve the repair bandwidth compared to repairing multiple failures sequentially.

The former studies considered the communication cost between the nodes to be homogeneous. However, in a realistic system, the storage nodes may be geographically separated in their locations, and hence the communication cost of downloading data from a geographically close helper node may be quite different from that of downloading from a helper node that is further away geographically. The model of a clustered (or rack-aware) distributed storage system assumes that the nodes are grouped into several clusters (racks) and assumes that the communication cost of downloading data from a node within the same cluster (rack) (which is also called the host cluster below) is much cheaper than downloading data from another cluster (rack). In fact, as shown in [8,9,10], the clustered structure incurs a substantial cross-cluster (cross-rack) bandwidth. The cross-cluster (cross-rack) capacity available per node in the worst case is only 1/5 to 1/20 of the intra-cluster (intra-rack) capacity, which indicates that the cross-cluster (cross-rack) bandwidth is more expensive than the intra-cluster (intra-rack) bandwidth.

Many attempts [11,12,13,14,15,16,17,18,19,20,21,22,23,24,25] have been made to reduce the cross-cluster bandwidth, in which repairing a single failure has been considered. Following these works, cases of clustered structures with multiple failures in each cluster were considered in [26,27,28,29,30,31,32], where the failed nodes downloaded data from other clusters so that the cross-cluster bandwidth applied.

As said, the cross-cluster (cross-rack) bandwidth is more expensive than the intra-cluster (intra-rack) bandwidth, so considering repairing multiple failures within the host cluster without cross-cluster traffic is an economical choice. In this article, we address the problem of repairing multiple failures in clustered storage systems, where multiple failures happen in each cluster. A data collector can retrieve the data file by connecting to any *k* nodes (from multiple clusters). We suppose the system has no cross-cluster bandwidth, and the target is to minimize the repair bandwidth within the host cluster. The main contributions of this article can be summarized as follows:

(1) We first study the trade-off between the storage and repair bandwidth for clustered distributed storage systems for multiple failures with zero cross-cluster bandwidth using a conventional maximum-flow, min-cut analysis over an information flow graph;
(2) We calculate the parameter values for two extreme points on the trade-off curve, namely the minimum storage clustered collaborative repair (MSCCR) point and the minimum bandwidth clustered collaborative repair (MBCCR) point;
(3) We analyze the repair bandwidth performance using different system parameters at the MSCCR and MBCCR points;
(4) We also provide explicit constructions to optimize the two extreme points on the trade-off curve, which implies that our constructions are optimal in terms of the repair bandwidth.

This article is organized as follows. Section 2 discusses related works. In Section 3, we briefly describe the clustered collaborative repair problem and introduce the standard notion of an information flow graph for the clustered collaborative repair problem. In Section 4, we give the min-cut bound (an upper bound on the file size) by inspecting the corresponding information flow graph. In Section 5, the two extreme points on the storage bandwidth trade-off curve, i.e., the MSCCR point and the MBCCR point, are identified. In Section 6, we analyze the normalized repair bandwidth at the MSCCR point and the MBCCR point and provide some results of simulations. In Section 7, we give the explicit code constructions for the MSCCR point and the MBCCR point, respectively. Finally, we conclude this article in Section 8 and point out further directions for future research.

## 2. Related Works

### 2.1. Collaborative Regenerating Codes

To reduce the repair bandwidth (repair traffic) when multiple failures happen in DSSs, *collaborative regenerating codes* (CRCs), or precisely fully collaborative regenerating codes, were proposed in [5,6,7]. Multiple failures were repaired simultaneously. The repair bandwidth of collaborative regenerating codes includes the amount of data exchanged between the failed nodes and the amount of data downloaded from the helper nodes. It has been shown [5,6,7] that collaborative repair improves the repair bandwidth compared to that when repairing multiple failures sequentially. Many attempts [33,34,35,36,37,38,39] have been made to construct collaborative regenerating codes at the minimum storage regenerating point and the minimum bandwidth regenerating point.

A related variant of fully collaborative regenerating codes is called *partially collaborative regenerating codes* [40], which can be viewed as a generalization of the collaborative repair mechanism and the sequential repair mechanism by allowing a subset of failed nodes to join the repair process collaboratively. A few constructions at the MSR point and the MBR point for partially collaborative regenerating codes were proposed in [41,42,43]. The problem of repairing multiple node failures has also been studied under the scenario of *centralized repair* [44,45], where a centralized node downloads data from some nodes that are still alive, computes the data, and then dispatches the data for all failed nodes.

### 2.2. Clustered Storage Codes

In a realistic system, the communication costs between storage nodes are heterogeneous when the nodes are geographically separated. The model of a clustered distributed storage system assumes that the nodes are grouped into several clusters. The model assumes that the communication cost of downloading data from a node within the same cluster is much cheaper than downloading data from another cluster.

A case with two clusters was studied in [11,12], and an upper bound on the file size that could be stored was derived. An explicit construction of maximum distance separable (MDS) array codes of rate 1/2 for a two-cluster case was proposed in [14]. A general case with more than two clusters was later studied in [13,14], where a single failed node was repaired by downloading data from the nodes in the host cluster and also from nodes in other clusters directly. An upper bound on the file size was proposed [13], and the existence of optimal repair codes was established via an information flow graph.

Recently, many attempts [15,16,17,18,19,20,21,22,23,24,25] have been made to reduce the cross-cluster bandwidth. In these works, a centralized node (also called a relay node/a compute unit) exists in each cluster, which collects data from the nodes within the cluster and then computes and dispatches data to repair the failed node. The failed node being repaired will download data from the centralized nodes in other clusters, indicating that the helpers are clusters rather than nodes. Hence, the model in these works can be seen as a centralized model, which is different from the model in [11,12,13,14] (which can be seen as a decentralized model), where the failed node being repaired directly downloads the data from nodes in other clusters without processing by the centralized node.

In [15], Hu et al. considered a clustered system with centralized nodes, and they derived an upper bound for the file size and showed the existence of minimum storage codes with optimal repair for the model. In [18], Hou et al. studied codes not only for the minimum storage but also for the minimum bandwidth. In [19], they derived the lower bound on the amount of information that needed to be accessed to repair a single failed node using an arbitrary number of helper racks. In [20], they proposed codes by product-matrix method for the minimum storage. Chen and Barg [21] further proposed a family of minimum storage rack-aware regenerating codes for all admissible parameters but with a high level of sub-packetization. Hou et al. [22] then proposed a family of minimum storage rack-aware regenerating codes that improved the sub-packetization level.

Clustered structures with multiple failures in each cluster were considered in [26,27,28,29,30,31,32]. In particular, Abdrashitov et al. [26] studied the problem of multiple node failures occurring in one cluster, and in their model, the data reconstruction was realized by connecting to any *k* clusters rather than any *k* nodes. Gupta and Lalitha [27,28,29] studied the case of multiple node failures occurring in more than one cluster, allowing for collaboration among clusters, where a centralized node existed for each cluster, the same setting as that seen in [15,16,17,18,19,21,22,23,24,25] as mentioned, and then the collaboration happened among the clusters instead of the nodes. Multiple node failures in a single cluster were considered in [30,31,32].

To conclude, we consider the problem of repairing multiple node failures in a clustered distributed storage system without a centralized node. The node repair happens within the host cluster (for the case of zero cross-cluster bandwidth), which is the most desirable setting, as mentioned, i.e., the intra-cluster bandwidth is much cheaper than the cross-cluster bandwidth. Within the cluster, the bandwidth will be further improved by utilizing the properties of collaborative regenerating codes.

## 3. The System Model

In this section, we will describe the clustered collaborative repair problem formally and the associated information flow graph that represents the problem.

A data object of size *M* is stored across *n* nodes, which is equally distributed across *L* clusters with *l* nodes each. Every node has a storage capacity of α. In this clustered distributed storage system, any choice of *k* nodes allows for object retrieval, where 2≤l≤k<n (a case where l≥k will be equivalent to the case in [5,6]). The system triggers a repair process when *t* failures occur in one cluster, and the *t* failures are repaired collaboratively in each cluster. Each node being repaired will download a β amount of data from the l−t surviving nodes in its host cluster and exchange a $\beta^{\prime }$ amount of data with the other t−1 nodes being repaired. The repair bandwidth for each node is $\gamma =(l-t)\beta +(t-1)\beta^{\prime }$. For ease of reading, some of the frequently used notations are summarized in Table 1.

Following the information flow graph approach from the seminal work [4], we consider the corresponding information flow graph associated with this clustered collaborative repair problem, where the data flow from a source *S* to a data collector DC. Suppose that a data collector DC connects to a subset of *k* nodes that are involved in different clusters. Let $k_{r}$ denote the number of nodes connected by DC in cluster *r*, where r∈[L] (for any positive integer *a*, denote [a]={1,…,a}). As such, we have $k=\sum_{i=1}^{L}k_{r}$. In each cluster *r*, the connected $k_{r}$ nodes are involved in different phases of repair (each repair phase is a repair process of *t* nodes), where each phase *i* involves a group of $u_{i}^{r}$ nodes such that $1\leq u_{i}^{r}\leq t$. Let $u^{r}=(u_{1}^{r},\ldots ,u_{h_{r}}^{r})$, where $\sum_{i=1}^{h_{r}}u_{i}^{r}=\min\{k_{r},l-t\}$.

**Figure 1:**
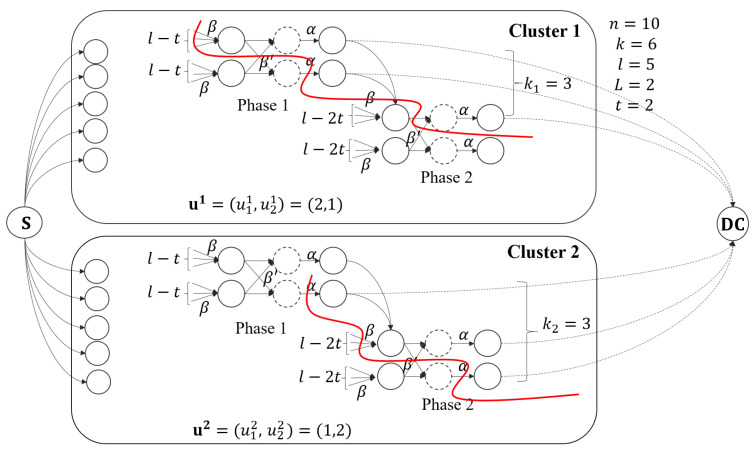
The information flow graph for n=10 nodes and L=2 clusters with k=6, $k_{1}=3$, and $k_{2}=3$. The red lines represent a possible cut on this flow graph.

We give an example of the information flow graph for two clusters here. As shown in Figure 1, the file is stored in n=10 nodes, which are divided into L=2 clusters, and each cluster has l=5 nodes. In each cluster, t=2 nodes are being repaired simultaneously. The data collector can retrieve the file by connecting to $k=k_{1}+k_{2}=6$ nodes, where $k_{1}=3$ nodes from cluster 1 in two phases with $u^{1}=(u_{1}^{1},u_{2}^{1})=(2,1)$, and $k_{2}=3$ nodes from cluster 2 in two phases with $u^{2}=(u_{1}^{2},u_{2}^{2})=(1,2)$.

## 4. Trade-Off Between the Storage Capacity and Repair Bandwidth

In this section, we derive a bound on the file size when the system parameters are fixed. The bound indicates the trade-off between the storage capacity α and the repair bandwidth γ.

By inspecting the information flow graph, as described in Section 3, we can obtain the following min-cut bound.

**Lemma** **1.** *The min-cut bound between the source S and the data collector DC is given by*

(1) $$\begin{array}{c} \min\;cut(S,DC)\geq \min_{\begin{array}{c} \sum_{r=1}^{L}k_{r}=k\\ k_{r}=0,\ldots ,l \end{array}}\sum_{r=1}^{L}\min_{u^{r}\in P^{r}}\sum_{i=1}^{h_{r}}u_{i}^{r}\min\{\alpha ,(l-t-\sum_{j=1}^{i-1}u_{j}^{r})\beta +(t-u_{i}^{r})\beta^{\prime }\}, \end{array}$$

*where*

$$\begin{array}{c} P^{r}=\{u^{r}=(u_{1}^{r},\ldots ,u_{h_{r}}^{r}):\;1\leq u_{i}^{r}\leq t\;\mathrm{and}\;\sum_{i=1}^{h_{r}}u_{i}^{r}=\min\{k_{r},l-t\}\}. \end{array}$$

The details of the examination for the cut in Lemma 1 are deferred to Appendix A. Here, we illustrate a possible cut in the information flow graph in Figure 1. The red lines in clusters 1 and 2 give a cut as an example. The contribution to the cut in cluster 1 is $(l-t)\beta +\beta^{\prime }+\alpha +\alpha =3\beta +\beta^{\prime }+2\alpha$, and the contribution to the cut in cluster 2 is $\alpha +(l-t-1)\beta +\beta^{\prime }+\alpha =2\alpha +2\beta +\beta^{\prime }$. So, this cut contains a $4\alpha +5\beta +2\beta^{\prime }$ amount of information.

According to the minimum cut–maximum flow theorem, we know that the file size *M* is bounded by the min-cut of the aforementioned information flow graph.

**Theorem** **1.** *A file of size M stored in a clustered distributed storage system with multiple failures and zero cross-cluster bandwidth must satisfy*

(2) $$\begin{array}{cc} M\leq & \min_{\begin{array}{c} \sum_{r=1}^{L}k_{r}=k\\ k_{r}=0,\ldots ,l \end{array}}\sum_{r=1}^{L}\min_{u^{r}\in P^{r}}\sum_{i=1}^{h_{r}}u_{i}^{r}\min\{\alpha ,(l-t-\sum_{j=1}^{i-1}u_{j}^{r})\beta +(t-u_{i}^{r})\beta^{\prime }\}, \end{array}$$

*where $P^{r}$ is the parameter defined in Lemma 1.*

An immediate corollary is as follows:

**Corollary** **1.** *When t=1, we have*

(3) $$M\leq \min_{\begin{array}{c} \sum_{r=1}^{L}k_{r}=k\\ k_{r}=0,\ldots ,l-1 \end{array}}\sum_{r=1}^{L}\sum_{i=1}^{k_{r}}\min\{\alpha ,(l-i)\beta \},$$

*which is the bound that can be derived from Theorem 1 in [13].*

**Figure 2:**
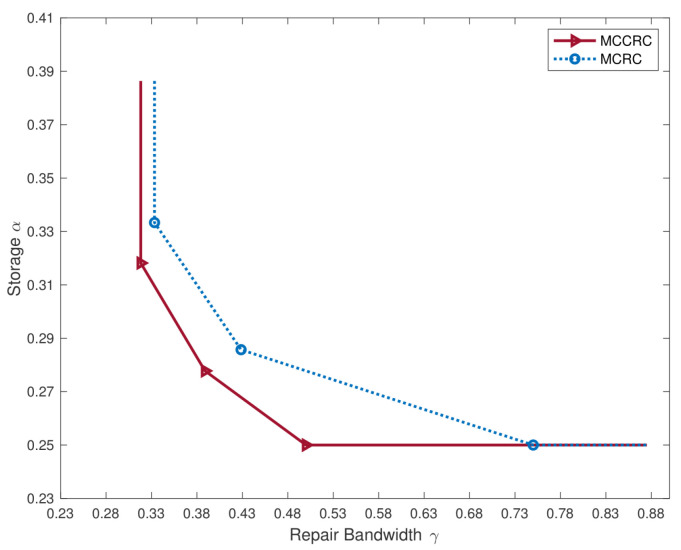
The trade-off between the storage capacity and the repair bandwidth for an MCCRC and an MCRC [13].

*An example:* The trade-off between the storage capacity and the repair bandwidth for clustered distributed storage systems equipped with the multi-clustered collaborative repair codes (MCCRCs) presented in this article is illustrated in Figure 2, compared with the trade-off for multi-clustered systems with a single failure to repair (i.e., systems equipped with multi-clustered repair codes (MCRCs)) [13]. The parameters in the example are specified as follows: the clustered system has n=10 nodes in L=2 clusters, each with a size of l=5, and any k=6 nodes can retrieve the file. t=2 nodes need to be repaired in each cluster, where 2 nodes are repaired simultaneously for the MCCRC, while 2 nodes are repaired separately and sequentially for the MCRC. An immediate observation from Figure 2 is that the MCCRC employing collaboration further reduces the repair bandwidth compared to that with an MCRC when repairing multiple failures.

## 5. Two Extreme Points

From Theorem 1, we can see that there are two special cases where the equality holds, namely the minimum storage clustered collaborative repair (MSCCR) point and the minimum bandwidth clustered collaborative repair (MBCCR) point. The values of the parameters in these two special cases are as follows.

*The minimum storage clustered collaborative repair (MSCCR) point:* Let $\sigma =\lfloor \frac{k}{l}\rfloor$, ⌊·⌋ be the floor function; the parameters of the minimum storage clustered collaborative repair (MSCCR) point on the trade-off curve have the following values:

(4) $$\begin{array}{cc} \; & \alpha =\frac{M}{\sigma (l-t)+\min\{k-\sigma l,l-t\}},\\ \; & \gamma =\frac{l-1}{t}\frac{M}{\sigma (l-t)+\min\{k-\sigma l,l-t\}},\\ \; & \beta =\beta^{\prime }=\frac{1}{t}\frac{M}{\sigma (l-t)+\min\{k-\sigma l,l-t\}}. \end{array}$$

*The minimum bandwidth clustered collaborative repair (MBCCR) point:* Let $\sigma =\lfloor \frac{k}{l}\rfloor$, parameters of the minimum bandwidth clustered collaborative repair (MBCCR) point on the trade-off curve have the following values:

(5) $$\begin{array}{cc} \; & \alpha =\gamma =\frac{M(2l-t-1)}{\sigma l(l-t)+\min\{k-\sigma l,l-t\}(2l-t-\min\{k-\sigma l,l-t\})},\;\\ \; & \beta =2\beta^{\prime }=\frac{2M}{\sigma l(l-t)+\min\{k-\sigma l,l-t\}(2l-t-\min\{k-\sigma l,l-t\})}. \end{array}$$

The details on identifying the parameters for the MSCCR point and the MBCCR point can be found in Appendix B.

## 6. Bandwidth Analysis

To understand the effect of the different values of the parameters on the repair bandwidth, we consider the normalized repair bandwidth $\bar{\gamma }=\gamma /M$. We will compute the value of the normalized repair bandwidth for different *l* values, the number of nodes in a cluster, at the minimum storage clustered collaborative repair (MSCCR) point and the minimum bandwidth clustered collaborative repair (MBCCR) point, respectively. It is shown that the normalized repair bandwidth is not monotonic with respect to *l* at the MSCCR point or the MBCCR point, respectively. Furthermore, we show that the performance of $\bar{\gamma }$ at the MSCCR and MBCCR points is different.

### 6.1. The MSCCR Point

The normalized repair bandwidth for the minimum storage is

(6) $$\begin{array}{cc} \bar{\gamma } & =\frac{l-1}{t\sigma (l-t)+t\min\{k-\sigma l,l-t\}}\\ \; & =\left\{\begin{array}{cc} \frac{l-1}{t(k-t)} & \frac{k+t}{2}\leq l\leq k\\ \frac{l-1}{2t(l-t)} & \frac{k}{2}\leq l\leq \frac{k+t}{2}\\ \;\;\;\vdots & \\ \frac{l-1}{t(k-it)} & \frac{k+t}{i+1}\leq l\leq \frac{k}{i}\\ \frac{l-1}{(i+1)t(l-t)} & \frac{k}{i+1}\leq l\leq \frac{k+t}{i+1}\\ \;\;\;\vdots \end{array}\right. \end{array}$$

where *i* is an integer, and i=1,2,…,σ.

It is shown that the normalized repair bandwidth $\bar{\gamma }$ at the MSCCR point is not monotonic with respect to the cluster size *l*. Specifically, on the interval $\left[\frac{k+t}{i+1},\frac{k}{i}\right]$, $\bar{\gamma }$ is increasing with respect to *l*. On the interval $\left[\frac{k}{i+1},\frac{k+t}{i+1}\right]$, $\bar{\gamma }$ is a decreasing function. The normalized repair bandwidth $\bar{\gamma }$ is a piecewise monotonic function with respect to *l*.

**Figure 3:**
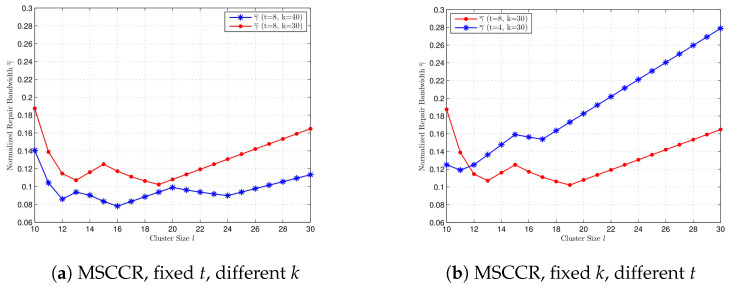
Comparison of normalized repair bandwidth at MSCCR point for cluster size l=10,…30, with different *k* and *t*, respectively. (**a**) t=8, k=40, and k=30. (**b**) k=30, t=8, and t=4.

In Figure 3, we compare the normalized repair bandwidth at the MSCCR point with different *k* and *t* values, where l=10,11,…,30. Since l|n, we can select n=30!. In Figure 3a, we compare $\bar{\gamma }$ for k=30 and k=40 when t=8. When *t* is fixed, a bigger value for *k* gives a smaller repair bandwidth at the MSCCR point for every l=10,…,30. In Figure 3b, we compare $\bar{\gamma }$ for t=8 and t=4 when k=30. When *k* is fixed, a bigger value for *t* not always gives a bigger $\bar{\gamma }$ but gives a smaller $\bar{\gamma }$ for the majority of *l*.

### 6.2. The MBCCR Point

The normalized repair bandwidth for the minimum storage is

(7) $$\begin{array}{cc} \bar{\gamma } & =\frac{2l-t-1}{l\sigma (l-t)+\min\{k-\sigma l,l-t\}(2l-t-\min\{k-\sigma l,l-t\})}\\ \; & =\left\{\begin{array}{cc} \frac{2l-t-1}{l(l-t)+(k-l)(3l-k-t)} & \frac{k+t}{2}\leq l\leq k\\ \frac{2l-t-1}{2l(l-t)} & \frac{k}{2}\leq l\leq \frac{k+t}{2}\\ \;\;\;\;\vdots & \\ \frac{2l-t-1}{il(l-t)+(k-il)((i+2)l-k-t)} & \frac{k+t}{i+1}\leq l\leq \frac{k}{i}\\ \frac{2l-t-1}{(i+1)l(l-t)} & \frac{k}{i+1}\leq l\leq \frac{k+t}{i+1}\\ \;\;\;\;\vdots \end{array}\right. \end{array}$$

where *i* is an integer, and i=1,2,…,σ.

We found that the normalized repair bandwidth $\bar{\gamma }$ at the MBCCR point was not monotonic with respect to the cluster size *l*. Specifically, on the interval $\left[\frac{k}{i+1},\frac{k+t}{i+1}\right]$, $\bar{\gamma }$ is a decreasing function. However, on the interval $\left[\frac{k+t}{i+1},\frac{k}{i}\right]$, $\bar{\gamma }$ is not a monotonic function with respect to *l*. Moreover, the normalized repair bandwidth $\bar{\gamma }$ at the MBCCR point is not a piecewise monotonic function with respect to *l*, which is different from the case at the MSCCR point.

**Figure 4:**
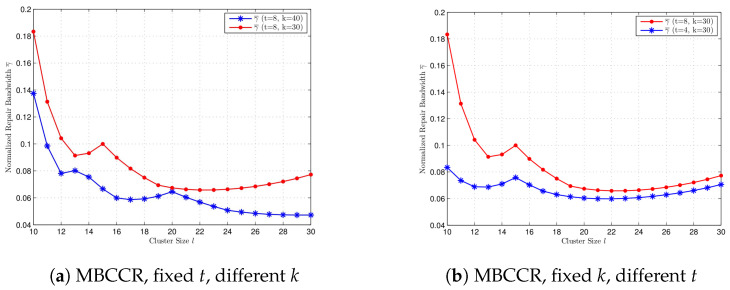
Comparison of the normalized repair bandwidth at the MBCCR point for cluster size l=10,…30, with different *k* and *t*, respectively. (**a**) t=8, k=40, and k=30. (**b**) k=30, t=8, and t=4.

In Figure 4, we compare the normalized repair bandwidth at the MBCCR point with different values for *k* and *t*, where l=10,11,…,30. We select n=30!. In Figure 4a, we compare $\bar{\gamma }$ for k=30 and k=40 when t=8. When *t* is fixed, a bigger value for *k* gives a smaller repair bandwidth at the MBCCR point for every l=10,…,30. In Figure 4b, we compare $\bar{\gamma }$ for t=8 and t=4 when k=30. When *k* is fixed, a bigger value for *t* gives a bigger value for $\bar{\gamma }$ for every l=10,…,30. It is shown that the performance of $\bar{\gamma }$ at the MBCCR point with different values of *t* is different from the case at the MSCCR point.

**Figure 5:**
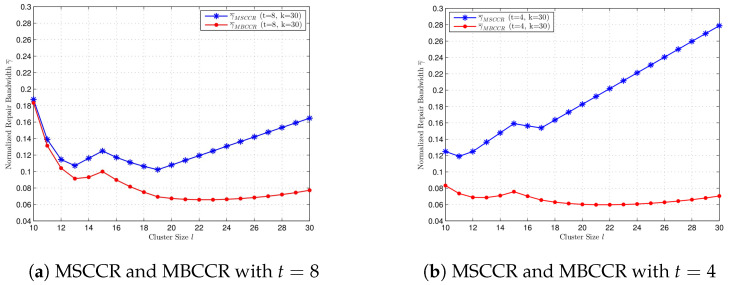
Comparison of normalized repair bandwidth at MSCCR point and MBCCR point. (**a**) t=8, k=30. (**b**) t=4, k=30.

Moreover, we compare the normalized repair bandwidth at the MSCCR point and the MBCCR point. In Figure 5a, we compare $\bar{\gamma }$ at the MSCCR point and the MBCCR point with k=30 and t=8. In Figure 5b, we compare $\bar{\gamma }$ at the MSCCR point and the MBCCR point with k=30 and t=4. Obviously, with the same system parameters, $\bar{\gamma }$ at the MSCCR point is bigger than that at the MBCCR point. When the number of failures *t* changes, the lower *t* is, the greater the difference in $\bar{\gamma }$ between the MSCCR point and the MBCCR point. In other words, when *k*, *l* is fixed, fewer node failures occur, and the greater the benefit to the repair bandwidth of using MBCCR codes.

## 7. Code Constructions

In this section, we propose two constructions satisfying the conditions of the MBCCR point and the MSCCR point, respectively, with the parameters n=L·l, l<k<n. We use two layers of codes for encoding; the outer layer uses a maximum rank distance (MRD) code, for example, a Gabidulin code. We will introduce MRD codes and Gabidulin codes first.

**Definition** **1.** *An [N,K,D] rank metric code $C\subseteq F_{q^{p}}^{N}$ (where q is a prime) is a linear code over $F_{q^{p}}$ of the length N, the dimension K, and the maximum rank D [46,47]. A rank metric code that attains a Singleton-like bound D≤N−K+1 on the rank metric is called a maximum rank distance (MRD) code.*

Here, the rank distance between two vectors $v_{1},v_{2}\in F_{q^{p}}^{N}$ is defined as $d(v_{1};v_{2})=Rk(v_{1}-v_{2}|F_{q})$, where $Rk(v_{1}-v_{2}|F_{q})$ is the maximum number of linearly independent coordinates of $v_{1}-v_{2}$ over the base field $F_{q}$, for a given basis of $F_{q^{p}}$ over $F_{q}$. Codes utilizing this distance metric are referred to as rank metric codes.

**Definition** **2.** *An [N,K,D] rank metric code over the extension field $F_{q^{p}}$ achieving the maximum rank distance D=N−K+1 (for p≥N) can be constructed using the following linearized polynomial:*

(8) $$f(x)=\sum_{i=0}^{K-1}u_{i}x^{q^{i}}.$$

*This is referred to as a Gabidulin code. A codeword in a Gabidulin code is defined as*

(9) $$c=(f(x_{1}),f(x_{2}),\ldots ,f(x_{N}))\in F_{q^{p}}^{N},$$

*with the coefficients given by the information message, and $x_{1},\ldots ,x_{N}\in F_{q^{p}}$ are linearly independent over $F_{q}$.*

We remark that a linearized polynomial f(·) satisfies $f(a_{1}x_{1}+a_{2}x_{2})=a_{1}f(x_{1})+a_{2}f(x_{2})$ for any $a_{1},a_{2}\in F_{q}$ and $x_{1},x_{2}\in F_{q^{p}}$. Given the evaluations of f(·) at any *K* linearly independent (over $F_{q}$) point in $F_{q^{p}}$, one can recover f(·) and therefore reconstruct the message vector $u_{0},\ldots ,u_{K-1}$ by performing polynomial interpolation (called the MDS property of Gabidulin code in the following content). These properties will be utilized in our code constructions.

### 7.1. Minimum Bandwidth Clustered Collaborative Repair (MBCCR) Codes

Consider a file of size $M=\sigma (l-t)l+(2l-t)\min\{k-\sigma l,l-t\}-\min{\{k-\sigma l,l-t\}}^{2}$ (the file can be seen as a data object vector of the length *M*), where σ=⌊k/l⌋, with coefficients in the finite field $F_{q^{p}}$ and *q* being a prime. Encoding the object using an [N,M,D] Gabidulin code, N=L(l−t)l and p≥N. The codeword $c=(c_{1},\ldots ,c_{N})$ is first divided into *L* groups denoted by $M_{r}$, r∈[L], with each being (l−t)l in size.

Rewriting each $M_{r}$, r∈[L] as the matrix form,

(10) $$M^{r}={\left[A^{r},B^{r}\right]}_{(l-t)\times l},$$

where $A^{r}$ is an (l−t)×(l−t) matrix. For each r∈[L], let $\Phi^{r}=\left[\phi_{1}^{r},\ldots ,\phi_{l}^{r}\right]$ be an (l−t)×l matrix with the entries in $F_{q}$ satisfying the principles that any l−t columns of $\Phi^{r}$ are linearly independent, $\Psi^{r}=\left[\psi_{1}^{r},\ldots ,\psi_{l}^{r}\right]$ is an invertible l×l matrix with the entries in $F_{q}$, and any (l−t)×(l−t) sub-matrix of $\Psi^{r}$ is invertible. $\Phi^{r}$ and $\Psi^{r}$ can be constructed using a Vandermonde matrix.

**Data placement in each cluster:** In each cluster *r*, r∈[L], node *i*, i∈[l] stores ${(\phi_{i}^{r})}^{T}M^{r}$ and $M^{r}\psi_{i}$. Since $({(\phi_{i}^{r})}^{T}M^{r})\psi_{i}-{(\phi_{i}^{r})}^{T}(M^{r}\psi_{i})=0$, node *i* stores α=2l−t−1 symbols.

**The repair of *t* failures in each cluster:** In each cluster *r*, r∈[L], without a loss of generality, suppose that the first *t* nodes fail. Each failed node *i*, i∈[t] downloads ${(\phi_{j}^{r})}^{T}M^{r}\psi_{i}$ and ${(\phi_{i}^{r})}^{T}M^{r}\psi_{j}$ from the nodes that are still alive *j*, j=t+1,…l. Since any l−t columns of $\Phi^{r}$ are linearly independent, the node *i* obtains $M^{r}\psi_{i}$; furthermore, it can compute ${(\phi_{i}^{r})}^{T}M_{r}\psi_{i}$. The total number of symbols downloaded from the helper nodes to one new node in the download phase of the repair process is β=2.

The missing ${(\phi_{i}^{r})}^{T}M^{r}$ can be obtained by contacting the other t−1 nodes being repaired. Node *i* contacts nodes $i^{\prime }\in \left[t\right]\setminus \{i\}$ and downloads ${(\phi_{i}^{r})}^{T}M_{r}\psi_{i^{\prime }}^{r}$ from each, and then node *i* obtains ${\{{(\phi_{i}^{r})}^{T}M_{r}\psi_{j}^{r}\}}_{j\in [l]}$. Since $\Psi^{r}$ is invertible, node *i* can obtain ${(\phi_{i}^{r})}^{T}M^{r}$. The total number of symbols downloaded during the collaboration process is t(t−1), that is, $\beta^{\prime }=1$; thus, γ=2(l−t)+(t−1)=2l−t−1.

**Data retrieval:** When any *k* nodes are connected by a DC, at least σ(l−t)l+(2l−t)$\min\{k-\sigma l,l-t\}-\min{\{k-\sigma l,l-t\}}^{2}$ linearly independent symbols over $F_{q}$ in $F_{q^{p}}$ can be obtained, where σ=⌊k/l⌋. Indeed, in each cluster *r*, although the storage capacity is 2l−t−1, each cluster only contains l(l−t) linearly independent symbols since only l(l−t) linearly independent symbols in $F_{q^{p}}$ are coded. For the *k* nodes being contacted, the fewer clusters they are in, the fewer independent symbols can be obtained. When σ=⌊k/l⌋, at least σ+1 clusters are being contacted, and we denote these clusters as cluster 1,…,σ,σ+1. We find that when all the nodes of σ (out of σ+1) clusters are being contacted by the DC, the DC will obtain the fewest linearly independent symbols. Therefore, without a loss of generality, suppose that all of the nodes in clusters 1,…,σ are being contacted; then, σ(l−t)l linearly independent symbols are obtained from cluster 1,…,σ. In the cluster σ+1, only k−σl nodes are being contacted. Here, we notice that in each cluster, l−t nodes are able to repair *t* failures; thus, at most (l−t)α=(l−t)(2l−t−1) symbols are linearly independent in each cluster. So, from k−σl nodes, only min{k−σl,l−t}(2l−t−min{k−σl,l−t}) linear independent symbols are obtained since the construction has the property $({(\phi_{i}^{r})}^{T}M^{r})\psi_{j}={(\phi_{i}^{r})}^{T}(M^{r}\psi_{j})$ for i≠j in the same cluster *r*. Therefore, from any *k* nodes of the system, at least σ(l−t)l+(2l−t)min{k−σl,${l-t\}-\min\{k-\sigma l,l-t\}}^{2}$ linearly independent symbols in $F_{q^{p}}$ are obtained, and they are all the codeword symbols of the outer Gabidulin code. Accordign to the MDS property of Gabidulin codes, any $\sigma (l-t)l+(2l-t)\min\{k-\sigma l,l-t\}-\min{\{k-\sigma l,l-t\}}^{2}=M$ symbols are linearly independent and are able to retrieve the data object. Therefore, from any *k* nodes, the DC can obtain at least $\sigma (l-t)l+(2l-t)\min\{k-\sigma l,l-t\}-\min{\{k-\sigma l,l-t\}}^{2}$ linearly independent symbols so that the file is retrieved.

The parameters of the construction are α=2l−t−1, $\beta =2,\beta^{\prime }=1,\gamma =2l-t-1$, which satisfy the condition at the MBCCR point when M=σ(l−t)l+min{k−σl,l−t}(2l−t−min{k−σl,l−t}.

In the MBCCR construction, we consider the information symbols over the finite field $F_{q}$. There are two layers of codes in the constructions. The outer code is an [N,M,D] Gabidulin code over $F_{q^{p}}$, with p≥N and N=L(l−t)t. The inner code is a product-matrix code over $F_{q}$. The node size is mainly determined by the outer Gabidulin code. Each symbol in $F_{q^{p}}$ can be seen as the vector over the field $F_{q}$ of the length p≥N, without a loss of generality. Let p=N; so, the node size for the MBCCR code is (2l−t−1)N. Furthermore, (2l−t−1)N=(2l−t−1)L(l−t)t scales as $O(n^{3})$, if we fix the number of clusters *L* to a relatively small number compared with the number of nodes *n*.

### 7.2. Minimum Storage Clustered Collaborative Repair (MSCCR) Codes

Consider a data object of size M=σ(l−t)t+tmin{k−σl,l−t} with coefficients in the finite field $F_{q^{p}}$, where σ=⌊k/l⌋. Encoding the object of size *M* by an [N,M,D] Gabidulin code, with N=L(l−t)t. The codeword $c=(c_{1},\ldots ,c_{N})$ is first divided into *L* groups, denoted by $M_{r}$, r∈[L], with each being (l−t)t in size.

Rewriting each $M_{r}$, r∈[L] as a t×(l−t) matrix $M^{r}$,

(11) $$M^{r}={\left[\begin{array}{c} m_{1}^{r}\\ \vdots \\ m_{t}^{r} \end{array}\right]}_{t\times (l-t)},$$

where $m_{i}^{r}$ is a row vector of size l−t. For each r∈[L], let $G^{r}=\left[g_{1}^{r},\ldots ,g_{l}^{r}\right]$ be a generator matrix of an (l,l−t) MDS code over $F_{q}$, where $g_{i}^{r}\in F_{q}^{l-t}$, for i∈[l].

**Data placement in each cluster:** In each cluster *r*, r∈[L], node *i* stores α=t symbols $M^{r}g_{i}^{r}$, where

(12) $$M^{r}g_{i}^{r}={\left[\begin{array}{c} m_{1}^{r}g_{i}^{r}\\ \vdots \\ m_{t}^{r}g_{i}^{r} \end{array}\right]}_{t\times 1},$$

**Repair of *t* failures in each cluster:** In each cluster *r*, r∈[L], when *t* nodes fail, without a loss of generality, we label *t* nodes from 1 to *t*. Then, these *t* nodes each contact the l−t nodes that are still alive; say the *i*-th node among these *t* failed nodes connects to nodes $i_{1},\ldots ,i_{l-t}$ and downloads $m_{i}^{r}g_{i_{j}}^{r}$ from the alive nodes $i_{j}$, j=1,…,l−t. Using the MDS property of $G^{r}$, node *i* can obtain $m_{i}^{r}$ and compute $m_{i}^{r}g_{i}^{r}$. The total number of symbols downloaded from the helper nodes to one node in the download phase of the repair process is β=1.

The *i*th node contacts nodes $i^{\prime }$, $i^{\prime }\in \left[t\right]\setminus \{i\}$, and downloads $m_{i^{\prime }}^{r}g_{i}^{r}$ to obtain the missing t−1 symbols. The total number of symbols downloaded during the collaboration process is t(t−1), that is $\beta^{\prime }=1$; thus, γ=(l−t)+(t−1)=l−1.

**Data retrieval:** When any *k* nodes are connected by a DC, at least σ(l−t)t+tmin{k−σl,l−t} linearly independent symbols (over $F_{q}$) in $F_{q^{p}}$ are obtained, where σ=⌊k/l⌋. Indeed, in each cluster *r*, only t(l−t) linearly independent symbols in $F_{q^{p}}$ are coded and stored. For the *k* nodes being contacted, the fewer clusters they are in, the fewer linear independent symbols are obtained. When σ=⌊k/l⌋, at least σ+1 clusters are contacted, and we denote these clusters as cluster 1,…,σ,σ+1. Without a loss of generality, suppose that all of the nodes in clusters 1,…,σ are being contacted; then, σ(l−t)t linearly independent symbols are obtained. In the cluster σ+1, only k−σl nodes are being contacted, and then tmin{k−σl,l−t} linear independent symbols are obtained. Therefore, from any *k* nodes, at least σ(l−t)t+tmin{k−σl,l−t} linearly independent symbols are obtained, and they are all of the codeword symbols of the outer Gabidulin code. According to the MDS property of Gabidulin codes, any σ(l−t)t+tmin{k−σl,l−t} symbols are linearly independent and are able to retrieve the object. Therefore, from any *k* nodes, the DC can obtain at least σ(l−t)t+tmin{k−σl,l−t} linearly independent symbols so that the file is retrieved.

The parameters of the construction are α=t, $\beta =2,\beta^{\prime }=1,\gamma =l-1$, which satisfy the condition of the MSCCR point when M=σ(l−t)t+tmin{k−σl,l−t}.

In the MSCCR code construction, the outer code is a Gabidulin code over $F_{q^{p}}$ with p≥N=L(l−t)t, and the inner code is an [l,l−t]-MDS code over $F_{q}$. Similarly to the discussion for MBCCR codes, we know that the node size for MSCCR code is tN. Moreover, tN=tL(l−t)t can be scaled as $O(n^{3})$ if *L* is a relatively small number compared with *n*.

We give a simple example of an MSCCR code below to illustrate the construction.

**Example** **1.** *Consider a system with its parameters as shown in Figure 1. The parameters have the values n=10,L=2,l=5,t=2,k=6, σ=⌊k/l⌋=1, M=σ(l−t)t+tmin{k−σl,l−t}=8.*

*We encode the object using an [12,8,5] Gabidulin code. The obtained codeword $c=(c_{1},\ldots ,c_{12})$ is first divided into two groups denoted by $M_{r}$, r=1,2, with each being 6 in size.*

*Rewriting each $M_{r}$ (r=1,2), as a 2×3 matrix $M^{r}$, that $M^{r}={\left[{(m_{1}^{r})}^{T},{(m_{2}^{r})}^{T}\right]}^{T}$. For each r=1,2, let $G^{r}=\left[g_{1}^{r},\ldots ,g_{5}^{r}\right]$ be a generator matrix of an (5,3) MDS code over $F_{q}$, where $g_{i}^{r}\in F_{q}^{3}$, for i∈[5]. In each cluster r=1,2, node i stores α=2 symbols $m_{1}^{r}g_{i}^{r}$ and $m_{2}^{r}g_{i}^{r}$.*

**Repair:** *In each cluster r=1,2, each failed node i, i=1,2 downloads $m_{i}^{r}g_{i_{j}}^{r}$ from the nodes that are alive $i_{j}$, j=1,…,3. Using the MDS property of $G^{r}$, the i-th failed node can obtain $m_{i}^{r}$ and compute $m_{i}^{r}g_{i}^{r}$. The total number of symbols downloaded from the helper nodes to one node in the download phase of the repair process is β=1. Then, the ith node contacts nodes $i^{\prime }\in \left[2\right]\setminus \{i\}$ and downloads $m_{i^{\prime }}^{r}g_{i}^{r}$ to obtain the missing 1 symbols. The total number of symbols downloaded during the collaboration process is 2, that is, $\beta^{\prime }=1$; thus, γ=(l−t)+(t−1)=4.*

**Data retrieval:** *When any six nodes are connected by a data collector, at least eight linearly independent symbols (over $F_{q}$) in $F_{q^{p}}$ are obtained. Indeed, in each cluster r=1,2, only six symbols in $F_{q^{p}}$ are linearly independent. When six nodes are being contacted, with five from the first cluster and one from the second cluster, the DC will obtain the fewest linearly independent symbols. Specifically, six linearly independent symbols in $F_{q^{p}}$ are obtained from the first cluster, and from the second cluster, only two linear independent symbols are obtained, totalling eight linearly independent symbols over $F_{q^{p}}$ that are obtained, and they are all the codeword symbols of the outer Gabidulin code. According to the MDS property of Gabidulin codes, any eight linearly independent symbols over $F_{q}$ in $F_{q^{p}}$ are able to retrieve the object. Therefore, from any six nodes, the data collector can obtain at least eight linearly independent symbols, and then the file is retrieved.*

**Figure 6:**
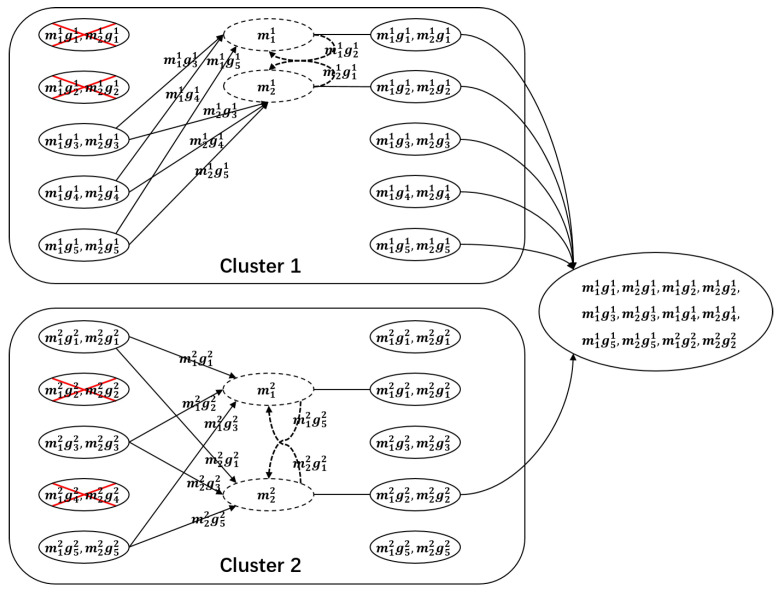
For a clustered distributed storage system with the parameters n=10, k=6, t=2, L=2, and l=5. In each cluster r=1,2, node *i* stores α=2 symbols $m_{1}^{r}g_{i}^{r},m_{2}^{r}g_{i}^{r}$. Each failed node downloads β=1 symbol from each of the alive nodes within the host cluster and exchanges $\beta^{\prime }=1$ symbol with another.

Figure 6 *illustrates a possible scenario where in each cluster, t=2 nodes fail. In cluster r=2, nodes 2 and 4 fail. The failed node 2 can be seen as the first failed node, and then it downloads $m_{1}^{2}g_{j}^{2}$ from the nodes that are still alive j=1,3,5. The failed node 4 can be seen as the second failed node and downloads $m_{2}^{2}g_{j}^{2}$ from the alive nodes j=1,3,5. When k=6 nodes are connected, 5 nodes from cluster 1 and 1 node from cluster 2, we obtain 12 symbols in $F_{q^{p}}$. For example, in the second cluster, the fourth node is contacted, and then we obtain twelve symbols $m_{1}^{1}g_{1}^{1},\ldots ,m_{1}^{1}g_{5}^{1},m_{2}^{1}g_{1}^{1},\ldots ,m_{1}^{1}g_{5}^{1},m_{1}^{2}g_{3}^{2},m_{1}^{2}g_{3}^{2}$ in $F_{q^{p}}$, and eight of them are linearly independent over $F_{q}$; then, according t the MDS property of the outer [12,8,5] Gabidulin code, the file can be retrieved.*

## 8. Conclusions

We have studied the repairing problem in a clustered distributed storage system, where each cluster is resilient to multiple failures within itself. A fundamental trade-off between the storage and repair bandwidth is derived by analyzing the associated information flow graph. Two extreme points on the trade-off curve, i.e., the MSCCR point and the MBCCR point, are studied. Their performance in terms of the repair bandwidth is studied as well. Furthermore, explicit and optimal code constructions for the two points are presented. We considered an ideal situation where the cross-cluster bandwidth is zero in this article. However, in a real clustered system, cross-cluster communication is possible, as stated in [8,9,10]. Therefore, a future direction for our study is to incorporate cross-cluster communication into the system. Moreover, since we proposed using Gabidulin codes as the outer codes for the optimal code constructions, which resulted in a larger node size, using other optimal or sub-optimal code constructions with a smaller node size would be another future direction.

## Figures and Tables

**Table 1:** Summary of notations.

Symbol	Meaning
*M*	size of the object
*n*	number of nodes storing data for one object
*L*	number of clusters
*l*	number of nodes in each cluster
*k*	reconstruction degree
*t*	number of failures which triggers a repair
α	storage capacity per node
β	amount of data downloaded from each survival node
$\beta^{\prime }$	amount of data downloaded from each new node
γ	total repair bandwidth per repair

## Data Availability

No new data were created or analyzed in this study.

## Appendix A

**Proof of Lemma 1.** We will adopt the following notations in the proof. In each cluster *r*, r∈[L], the repair nodes during the *i*th phase of repair are denoted by $x^{r,i,j}$, where *j* is the count of the nodes during the *i*th phase. Every node $x^{r,i,j}$ is seen as a logical triple $(x_{in}^{r,i,j},x_{coor}^{r,i,j},x_{out}^{r,i,j})$, formed of an incoming node, a collaborating node, and an output node, used to model the storage capacity and the collaborative process. Consider a data collector DC that connects to *k* output nodes corresponding to a set *K* of nodes from *L* clusters. For each cluster *r*, r∈[L], say $\{x_{out}^{r,i,j}:(i,j)\in K_{r}\}$, where $K=\cup_{r=1}^{L}K_{r}$, $|K_{r}|=k_{r}$, and $\sum_{r=1}^{L}k_{r}=k$. We show that any cut C between *S* and DC in the graph has a capacity that satisfies (1). We can assume that all of the edges of the data collector have an infinite capacity, so we only need to consider the cut $(U,\bar{U})$, with S∈U and $\{x_{out}^{r,i,j}:(i,j)\in K,r\in \left[L\right]\}\subseteq \bar{U}$. Since the repairs are only carried out in the host cluster, we obtain $\{x_{out}^{r,i,j}:(i,j)\in K,r\in \left[L\right]\}=\cup_{r=1}^{L}\{x_{out}^{r,i,j}:(i,j)\in K_{r}\}\subseteq \bar{U}$, and we can calculate the cut in each cluster separately. Let $C_{r}$ denote the edges in cluster *r* in the cut, with cut C = $\sum_{r=1}^{L}C_{r}$. In each cluster *r*, we consider the following repair phases. **The first repair phase:** In each cluster *r*, r∈[L], let $J_{1}^{r}$ be the set of indices such that $\{x_{out}^{r,1,j}:j\in J_{1}^{r}\}$ is the first group of output nodes in cluster *r* in $\bar{U}$ corresponding to the first repair. The set contains $|\{x_{out}^{r,1,j}:j\in J_{1}^{r}\}|=u_{1}^{r}$ nodes. Consider a subset $I_{1}^{r}\subset J_{1}^{r}$ of size *m* such that $\{x_{in}^{r,1,j}:j\in I_{1}^{r}\}\subset U$ and $\{x_{in}^{r,1,j}:j\in J_{1}^{r}-I_{1}^{r}\}\subset \bar{U}$. Then, *m* can take values between 0 and $u_{1}^{r}$. Consider first the *m* nodes $\{x_{in}^{r,1,j}:j\in I_{1}^{r}\}\subset U$. For each node $x_{in}^{r,1,j}\in U$, either (1) $x_{coor}^{r,1,j}\in U$, and then edge $x_{coor}^{r,1,j}\to x_{out}^{r,1,j}\in C_{r}$, and the contribution to the cut is α, or (2) $x_{coor}^{r,1,j}\in \bar{U}$, and then $x_{in}^{r,1,j}\to x_{coor}^{r,1,j}\in C_{r}$, and the contribution to the cut is at least α. Consider next the $u_{1}^{r}-m$ other nodes $\{x_{in}^{r,1,j}:j\in J_{1}^{r}-I_{1}^{r}\}\subset \bar{U}$. For each node, the contribution comes from multiple sources. (1) The cut contains d=l−t edges carrying β coefficients: since $x_{out}^{r,1,j}$ are the first output nodes in $\bar{U}$, the edges come from the output nodes in *U*. (2) The cut contains at least $t-u_{1}^{r}+m$ edges carrying $\beta^{\prime }$ coefficients thanks to the coordination step: the node $x_{coor}^{r,1,j}$ has t−1 incoming edges $x_{in}^{r,1,j^{\prime }}\to x_{coor}^{r,1,j}$. However, $|\{x_{in}^{r,1,j^{\prime }}\})\cap \bar{U}|=u_{1}^{r}-m-1$, so the cut contains at least $(t-1)-(u_{1}^{r}-m-1)=t-u_{1}^{r}+m$ such edges carrying $\beta^{\prime }$ coefficients. Therefore, the total contribution of these nodes in the cut is $c_{1}^{r}(m)\geq m\alpha +(u_{1}^{r}-m)((l-t)\beta +(t-u_{1}^{r}+m)\beta^{\prime })$. Since the function $c_{1}^{r}$ is concave on the interval $\left[0,u_{1}\right]$, Jensen’s inequality yields

(A1) $$c_{1}^{r}(m)\geq u_{1}\min\{\alpha ,\;(l-t)\beta +(t-u_{1}^{r})\beta^{\prime }\}.$$

**The second repair phase:** Let $\{x_{out}^{r,2,j}:j\in J_{2}^{r}\}$ be the second group of output nodes in cluster *r* in $\bar{U}$ corresponding to the second repair. We repeat the same reasoning. We first consider the *m* nodes $\{x_{in}^{r,2,j}:j\in I_{2}^{r}\}\subset U$, where the contribution of each node is α. Then, we consider the $u_{2}^{r}-m$ nodes $\{x_{in}^{r,2,j}:j\in J_{2}^{r}-I_{2}^{r}\}\subset \bar{U}$. For each node, we have (1) the cut containing at least $l-t-u_{1}^{r}$ edges carrying β: since these $\{x_{in}^{r,2,j}\}$ are the second group of output nodes in $\bar{U}$, and at most $u_{1}^{r}$ edges come from the output nodes in $\bar{U}$, at least $l-t-u_{1}^{r}$ edges come from the output nodes in *U*; (2) similarly to the first phase, the cut contains at least $t-u_{2}^{r}+m$ edges carrying $\beta^{\prime }$. Thus, the total contribution of these nodes is $c_{2}^{r}(m)\geq u_{2}^{r}\min\{\alpha ,(l-t-u_{1}^{r})\beta +(t-u_{2}^{r})\beta^{\prime }\}$. In general, for the *i*th repair phase,

(A2) $$c_{i}^{r}(m)\geq u_{i}^{r}\min\{\alpha ,(l-t-\sum_{j=1}^{i-1}u_{j}^{r})\beta +(t-u_{i}^{r})\beta^{\prime }\}.$$

Therefore, in each cluster *r*, the contribution to the cut is

(A3) $$c^{r}\geq \min_{u^{r}\in P^{r}}\sum_{i=1}^{h_{r}}u_{i}^{r}\min\{\alpha ,(l-t-\sum_{j=1}^{i-1}u_{j}^{r})\beta +(t-u_{i}^{r})\beta^{\prime }\}.$$

where $\sum_{i=1}^{h_{r}}u_{i}^{r}=\min\{k_{r},l-t\}$. Notice that we have the restriction for $u_{i}^{r}$ because for $i\geq i_{0}$, where $i_{0}=\min\{i:\sum_{j=1}^{i-1}u_{i}^{r}\geq l-t\}$, the cut contains $u_{i}^{r}\min\{\alpha ,(t-u_{i}^{r})\beta^{\prime }\}$, but $(t-u_{i}^{r})\beta^{\prime }$ are all from the former phases; hence, when $i\geq i_{0}$, the contribution to the cut is 0. Therefore, we only need to count the contribution for *i* where $\sum_{j=1}^{i-1}u_{i}^{r}\leq l-t$. Since cut C = $\sum_{r=1}^{L}C_{r}$, summing the contributions in all of the clusters leads to bound (1). □

## Appendix B. The Parameters for the MSCCR Point and the MBCCR Point

### Appendix B.1. Parameters for the MSCCR Point

In each cluster, any l−t nodes are able to repair *t* failures so that each cluster has at most (l−t)α linearly independent information symbols; hence, for any *k* out of *n* nodes, at least σ(l−t)α+min{k−σl,l−t}α linearly independent information symbols can be obtained by the data collector, where σ=⌊k/l⌋. From the (n,k) recovery property, we have $\alpha \geq \frac{M}{\sigma (l-t)+\min\{k-\sigma l,l-t\}}$. The MSCCR point provides the lowest possible storage α while minimizing the repair bandwidth. Therefore, we minimize α first. We denote $k_{0}=\sigma (l-t)+\min\{k-\sigma l,l-t\}$ for simplicity, and then $\alpha =\frac{M}{k_{0}}$, and each component of the sum in (2) is at least equal to $\frac{M}{k_{0}}$; then,

(A4) $$(l-t-\sum_{j=1}^{i-1}u_{j}^{r})\beta +(t-u_{i}^{r})\beta^{\prime }\geq \frac{M}{k_{0}},\;\forall i\in \left[h_{r}\right],r\in \left[L\right].$$

We consider two particular patterns of the repair phases to help determine the minimum storage clustered collaborative repair point: (i) $u^{r}=\left[1,1,\ldots \right]$, for any r=1,2,⋯,L, and (ii) $u^{r}=\left[t,t,\ldots \right]$, for any r=1,2,⋯,L.

For pattern (i), $u^{r}=\left[1,1,\ldots \right]$, r∈[L], Equation (A4) corresponds to

(A5) $$(l-t-(i-1))\beta +(t-1)\beta^{\prime }\geq \frac{M}{k_{0}},\;\forall i\in \left[\min\{k_{r},l-t\}\right],$$

and thus

(A6) $$\beta^{\prime }\geq \frac{\frac{M}{k_{0}}-(l-t-i+1)\beta }{t-1},\;\forall i\in \left[\min\{k_{r},l-t\}\right].$$

Note that the right-hand side of Equation (A6) grows linearly with *i*; therefore,

(A7) $$\begin{array}{c} \beta^{\prime }\geq \frac{\frac{M}{k_{0}}-(l-t-\min\{k_{r},l-t\}+1)\beta }{t-1}, \end{array}$$

For pattern (ii), $u^{r}=\left[t,t,\ldots \right]$, r∈[L], Equation (A4) corresponds to

(A8) $$(l-it)\beta \geq \frac{M}{k_{0}},\;\forall i\in \left[\frac{\min\{k_{r},l-t\}}{t}\right],$$

Here, we assume $t|\min\{k_{r},l-t\}$. For a case where $t∤\min\{k_{r},l-t\}$, it can be reduced into the same condition with $t|\min\{k_{r},l-t\}$; hence, we omit the analysis here. From Equation (A8), we obtain

(A9) $$\beta \geq \frac{M}{k_{0}}\frac{1}{l-it},\;\forall i\in \left[\frac{\min\{k_{r},l-t\}}{t}\right],$$

which is equivalent to

(A10) $$\beta \geq \frac{M}{k_{0}}\frac{1}{l-\min\{k_{r},l-t\}}.$$

The repair bandwidth is

(A11) $$\begin{array}{c} \gamma =(l-t)\beta +(t-1)\beta^{\prime }\geq (\min\{k_{r},l-t\}-1)\beta +\frac{M}{k_{0}}, \end{array}$$

where the inequality of (A11) comes from Equation (A7). Equation (A11) implies that γ grows linearly with β; therefore, we take the smallest value of β, where $\beta =\frac{M}{k_{0}}\frac{1}{l-\min\{k_{r},l-t\}}$. Hence,

(A12) $$\begin{array}{c} \gamma \geq \frac{M}{k_{0}}+\frac{M}{k_{0}}\frac{\min\{k_{r},l-t\}-1}{l-\min\{k_{r},l-t\}}=\frac{M}{k_{0}}\frac{l-1}{l-\min\{k_{r},l-t\}}. \end{array}$$

Since (A12) holds for all $\min\{k_{r},l-t\}\leq l-t$, then we have the minimum values of α, γ, β, and $\beta^{\prime }$ as

(A13) $$\alpha =\frac{M}{k_{0}},\;\;\gamma =\frac{M}{k_{0}}\frac{l-1}{t},\;\;\beta =\beta^{\prime }=\frac{M}{k_{0}}\frac{1}{t}.$$

Note that the parameters obtained indeed satisfy (2). Each term in the second sum of (2) is at most $u_{i}^{r}\frac{M}{k_{0}}$, the minimum of the second sum value is equal to $\frac{k_{r}}{k_{0}}M$, and the minimum over $k_{r}$ of the first sum is equal to *M* when all of the terms are $u_{i}^{r}\frac{M}{k_{0}}$. Thus, each element of the second sum must satisfy

(A14) $$(l-t-\sum_{j=1}^{i-1}u_{j}^{r})\beta +(t-u_{i}^{r})\beta^{\prime }\geq \frac{M}{k_{0}}.$$

Replacing $\beta ,\beta^{\prime }$ with their values at the MSCCR point to obtain $l-\sum_{j=1}^{i}u_{i}^{r}\geq t,$ which is satisfied since $\sum_{j=1}^{i}u_{i}^{r}\leq \min\{k_{r},l-t\}\leq l-t$. Therefore, the MSCCR point has the values of the parameters in (4).

### Appendix B.2. Parameters for the MBCCR Point

The MBCCR point provides the lowest possible repair bandwidth γ while minimizing the storage α. Therefore, to optimize γ, we let α grow, and then each of the terms in (2), which contains β and $\beta^{\prime }$, is smaller than α, and the minimum value of the storage capacity $\alpha =\gamma =(l-t)\beta +(t-1)\beta^{\prime }$. Denote $\kappa =\{(k_{1},\ldots ,k_{L}):k_{r}=0,\ldots ,l,\;r\in \left[L\right],\;\sum_{r=1}^{L}k_{r}=k\}$.

For pattern (ii), $u^{r}=\left[t,t,\ldots \right]$, r=1,2,⋯,L. Here, we only consider $t|\min\{k_{r},l-t\}$ due to limited space; for a case where $t∤\min\{k_{r},l-t\}$, it can be reduced into the same result with $t|\min\{k_{r},l-t\}$. We denote $\Delta =\min\{k_{r},l-t\}$ for simplicity, and it is required that

(A15) $$\begin{array}{c} M\leq \min_{\kappa }\sum_{r=1}^{L}\sum_{i=1}^{\frac{\Delta }{t}}t(l-it)\beta =\min_{\kappa }\sum_{r=1}^{L}(l\Delta \beta -\frac{t}{2}\Delta \beta -\frac{1}{2}\Delta^{2}\beta ). \end{array}$$

Then, the equation is equivalent to

(A16) $$\beta \geq \frac{M}{\min_{\kappa }\sum_{r=1}^{L}(l\Delta -\frac{t}{2}\Delta -\frac{1}{2}\Delta^{2})}.$$

For pattern (i), $u^{r}=\left[1,1,\ldots \right]$, r∈[L], it is required that

(A17) $$\begin{array}{cc} M & \leq \min_{\kappa }\sum_{r=1}^{L}\sum_{i=1}^{\Delta }\left[(l-t-i+1)\beta +(t-1)\beta^{\prime }\right]\\ \; & =\min_{\kappa }\sum_{r=1}^{L}\left[(l-t)\Delta \beta -\frac{1}{2}\Delta^{2}\beta +\frac{1}{2}\Delta \beta +(t-1)\Delta \beta^{\prime }\right], \end{array}$$

which is equivalent to

(A18) $$\begin{array}{c} \beta^{\prime }\geq \frac{M-\min_{\kappa }\sum_{r=1}^{L}((l-t)\Delta -\frac{1}{2}\Delta^{2}+\frac{1}{2}\Delta )\beta }{(t-1)\min_{\kappa }\sum_{r=1}^{L}\Delta }. \end{array}$$

Thus, $\beta^{\prime }$ grows linearly with β, and we take the smallest value of β from Equation (A16):

(A19) $$\begin{array}{c} \beta^{\prime }\geq \frac{1}{2}\frac{M}{\min_{\kappa }\sum_{r=1}^{L}(l\Delta -\frac{t}{2}\Delta -\frac{1}{2}\Delta^{2})}. \end{array}$$

Therefore, the minimum values of α, γ, β, and $\beta^{\prime }$ are

(A20) $$\begin{array}{cc} \; & \alpha =\gamma =\frac{M(2l-t-1)}{\min_{\kappa }\sum_{r=1}^{L}(l\Delta -\frac{t}{2}\Delta -\frac{1}{2}\Delta^{2})}\\ \; & \beta =2\beta^{\prime }=\frac{M}{\min_{\kappa }\sum_{r=1}^{L}(l\Delta -\frac{t}{2}\Delta -\frac{1}{2}\Delta^{2})}. \end{array}$$

Let $\sigma =\lfloor \frac{k}{l}\rfloor$; it can be checked that

(A21) $$\begin{array}{c} \min_{\kappa }\sum_{r=1}^{L}(l\Delta -\frac{t}{2}\Delta -\frac{1}{2}\Delta^{2})=\frac{\sigma }{2}l(l-t)+\frac{1}{2}\min\{k-\sigma l,l-t\}(2l-t-\min\{k-\sigma l,l-t\}). \end{array}$$

Hence, we obtain the values in (5).

The values of the parameters are consistent with (2), which can be checked, similarly to what was applied to the parameters at the MSCCR point, by noticing that α is always larger than or equal to the repair bandwidth. Therefore, the MBCCR point has the values of the parameters in (5).
